# Overexpression of Exosomal Cardioprotective miRNAs Mitigates Hypoxia-Induced H9c2 Cells Apoptosis

**DOI:** 10.3390/ijms18040711

**Published:** 2017-03-28

**Authors:** Jinwei Zhang, Jideng Ma, Keren Long, Wanling Qiu, Yujie Wang, Zihui Hu, Can Liu, Yi Luo, Anan Jiang, Long Jin, Qianzi Tang, Xun Wang, Xuewei Li, Mingzhou Li

**Affiliations:** Institute of Animal Genetics and Breeding, College of Animal Science and Technology, Sichuan Agricultural University, Chengdu 611130, Sichuan, China; Jinweizhang50@163.com (J.Z.); jideng_ma@sina.com (J.M.); longkeren@163.com (K.L.); qiuwanling2016@163.com (W.Q.); wangyujie715@163.com (Y.W.); Huzihui2016@163.com (Z.H.); 18227550880@163.com (C.L.); luoyi670@163.com (Y.L.); lingdang317@163.com (A.J.); longjin8806@163.com (L.J.); wupie@163.com (Q.T.); xun_wang007@163.com (X.W.)

**Keywords:** H9c2 cells, microRNA, exosome, apoptosis, hypoxia

## Abstract

Recent evidence suggests that hypoxia caused by acute myocardial infarction can induce cardiomyocyte apoptosis. Exosomes are signalling mediators that contribute to intercellular communication by transporting cytosolic components including miRNAs, mRNAs, and proteins. However, the systemic regulation and function of exosomal miRNAs in hypoxic cardiomyocytes are currently not well understood. Here, we used small RNA sequencing to investigate the effects of hypoxia stress on miRNAome of rat cardiomyoblast cells (H9c2) and corresponding exosomes. We identified 92 and 62 miRNAs in cells and exosomes, respectively, that were differentially expressed between hypoxia and normoxia. Hypoxia strongly modulated expression of hypoxia-associated miRNAs in H9c2 cells, and altered the miRNAome of H9c2 cells-derived exosomes. Functional enrichment analysis revealed extensive roles of differentially expressed exosomal miRNAs in the HIF-1 signalling pathway and in apoptosis-related pathways including the TNF, MAPK, and mTOR pathways. Furthermore, gain- and loss-of-function analysis demonstrated potential anti-apoptotic effects of the hypoxia-induced exosomal miRNAs, including miR-21-5p, miR-378-3p, miR-152-3p, and let-7i-5p; luciferase reporter assay confirmed that *Atg12* and *Faslg* are targets of miR-152-3p and let-7i-5p, respectively. To summarize, this study revealed that hypoxia-induced exosomes derived from H9c2 cells loaded cardioprotective miRNAs, which mitigate hypoxia-induced H9c2 cells apoptosis.

## 1. Introduction

Acute myocardial infarction (AMI), a common consequence of coronary artery occlusion and myocardial ischemia, is one of the leading causes of death worldwide [1,2]. Hypoxia caused by AMI leads to cardiomyocyte apoptosis [3]. Unlike necrosis, apoptosis is a process of programmed cell death that is actively controlled by a series of genes to maintain homeostasis [4]. Hypoxia-inducible factor (HIF), which comprises α and β subunit, is a master regulator of cellular response to hypoxia [5]. Under hypoxic conditions, HIF-α (HIF-1α, HIF-2α and HIF-3α) is activated and translocates from the cytoplasm to the nucleus, where it dimerizes with HIF-β and binds to hypoxia response elements (HRE) in the promoters of HIF-controlled genes [6]. Afterward, hypoxia-adaptive pathways will be activated, including angiogenesis, anaerobic metabolism, erythropoiesis, and iron metabolism, to reduce oxygen consumption and increase cell survival [7]. Nonetheless, continuous hypoxic stress will eventually lead to apoptosis, or even necrosis [8].

miRNA, a class of endogenous non-coding RNA, is well established as a mediator of post-transcriptional regulation in a wide array of biological processes, including differentiation, proliferation and apoptosis [9]. Recently, a number of studies have demonstrated that hypoxia can modulate expression of a specific subset of miRNAs, termed “hypoxamiRs”; and that these hypoxamiRs coordinate HIF signaling to regulate cell proliferation, metabolism, DNA repair, and apoptosis [10]. Exosomes are nano-sized (50–200 nm) vesicles released by many cell types via exocytosis. They can mediate cell-to-cell communication and function as signalling agents by delivering nucleic acids (including miRNA and mRNA) and proteins between cells [11]. Emerging evidence indicates that exosome-mediated transfer of miRNAs between cardiomyocytes contributes to reconciling cardiac physiology and pathology state. Cardiac fibroblast-derived exosomes deliver miR-21* to cardiomyocytes to induce myocardial hypertrophy by targeting *SORBS2* and *PDLIM5* [12]. miR-21-5p and miR-210-3p are enriched in exosomes secreted from mouse cardiac fibroblast-derived induced pluripotent stem cells (iPS cells), and prevent H9c2 cells apoptosis by regulating Nanog and HIF-1α, respectively [13]. miR-210-3p derived from exosomes secreted by cardiac progenitor cells (CPCs) inhibits cardiomyocyte apoptosis by targeting *ephrin A3* and *PTP1b* [14]. However, the systemic regulation and function of exosomal miRNA in cardiomyocytes under AMI-induced hypoxic stress are poorly understood.

In this study, we established a model of anoxia using H9c2 cells, an immortal rat cardiomyoblast cell line, in hypoxic conditions that mimicked the hypoxia caused by AMI in vitro. We used small RNA sequencing to investigate the miRNA transcriptome of H9c2 cells and exosomes collected from hypoxia and normoxia. We found that expression of hypoxamiRs was strongly regulated by hypoxia in H9c2 cells; furthermore, hypoxia markedly altered the miRNAome of H9c2 cells-derived exosomes. The exosomal miRNAs that were differentially expressed (DE miRNAs) between hypoxia and normoxia were mainly involved in the HIF-1 signaling pathway and cell apoptosis. Our results reveal that exosomes produced by H9c2 cells in response to hypoxia contain cardioprotective miRNAs and mitigate H9c2 cells apoptosis after hypoxia, which may present a potential novel treatment for AMI and other types of heart disease.

## 2. Results and Discussion

### 2.1. Hypoxia Decreases Cell Viability and Induces Apoptosis in H9c2 Cells

**Figure 1:**
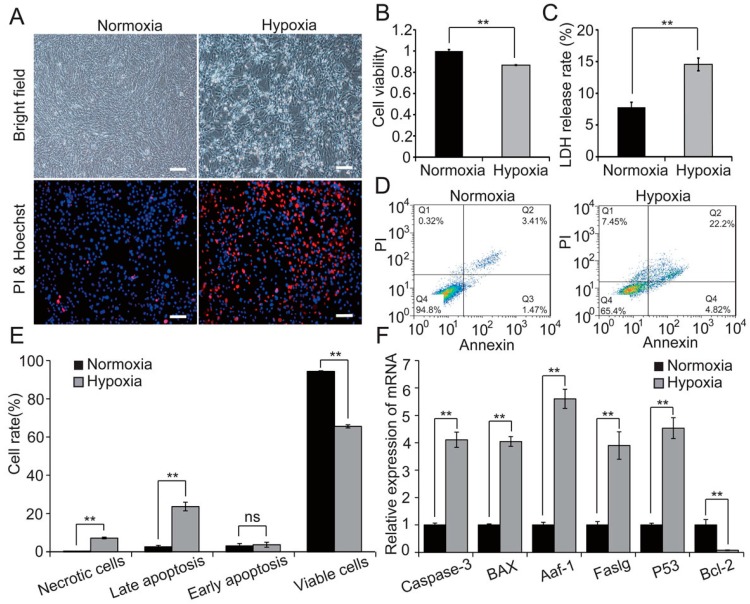
Hypoxia induced H9c2 cells apoptosis. (**A**) H9c2 cells were exposed to hypoxia or normoxia for 48 h then observed by bright-field microscopy, and by fluorescence microscopy with propidium iodide (PI) and Hoechst 33324 double-staining. Scale bar, 10 μm; cell viability (**B**), membrane integrity (**C**) and apoptosis rate (**D**,**E**) were evaluated by CCK8 assay, LDH release assay and flow cytometry analysis, respectively; and (**F**) expression levels of apoptosis-related genes were detected by qRT-PCR. Three independent experiments performed in triplicate; all data are expressed as mean ± SD. ** *p* < 0.01, ns: no significant.

A previous study demonstrated that hypoxia induced cardiomyocyte apoptosis, which was involved in the pathogenesis of AMI [15]. To illustrate the physiological effect of acute hypoxia on H9c2 cells, we cultured H9c2 cells in vitro under hypoxic conditions (1% O_2_) for 48 h. Hypoxia induced notable changes in cell morphology and growth, and induced H9c2 cell apoptosis (Figure 1A). Furthermore, CCK8 (Figure 1B) and flow cytometry analysis (Figure 1D,E) indicated that hypoxia significantly reduced H9c2 cell viability and induced apoptosis, in keeping with previous studies [16,17]. Additionally, we analysed cell membrane integrity by LDH release assay, which showed a higher LDH leakage rate in hypoxia compared with normoxia (Figure 1C). Furthermore, hypoxia markedly increased expression of the pro-apoptotic genes *Caspase-3*, *Bax*, *Aaf-1*, *Faslg*, and *P53*, whereas expression of the anti-apoptotic gene *Bcl-2* was inhibited by hypoxia (Figure 1F). These results indicate that, as expected, hypoxia induced H9c2 cells apoptosis.

### 2.2. Hypoxia Significantly Modulates hypoxamiR Levels in H9c2 Cells

**Figure 2:**
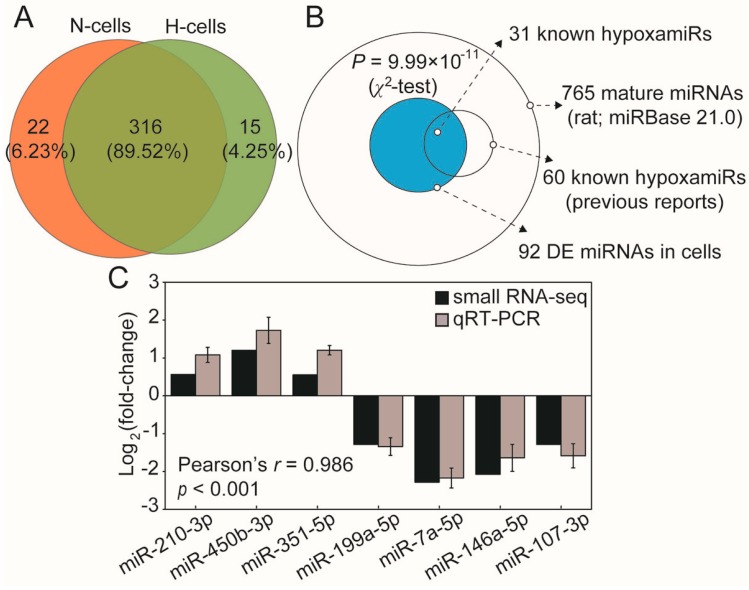
miRNA expression profiles of normoxic and hypoxic H9c2 cells. (**A**) Venn diagram showing the numbers of overlapping and unique miRNAs detected in normoxic and hypoxic H9c2 cells; (**B**) Distribution of miRNAs. Out of 765 rat mature miRNAs deposited in miRBase 21.0, 60 (7.84%) miRNAs have been designated as hypoxamiRs based on the previous reports [7,10], and the hypoxamiRs (31 of 92, 33.69%) were overrepresented in the DE miRNAs of cells after hypoxia (blue circle); and (**C**) Seven miRNAs were randomly selected for qRT-PCR validation. “H” and “N” represented “Hypoxia” and “Normoxia”, respectively.

Hypoxia modulates expression of hypoxamiRs, which can directly and indirectly regulate hypoxia-adaptive pathways to preserve cell viability [7]. To comprehensively explore hypoxia-induced variations in the miRNA transcriptome, H9c2 cells were cultured in normoxic and hypoxic conditions, then collected and prepared for Illumina small RNA-seq. We identified 338 and 331 known miRNAs in normoxic and hypoxic H9c2 cells, respectively. The number of overlapping and unique miRNAs in normoxic and hypoxic H9c2 cells is shown in Figure 2A. Overlapping miRNAs account for 89.5% of the total, which shows that hypoxia induced a small change in the miRNA species. Afterwards, 92 differentially-expressed miRNAs were identified in H9c2 cells after hypoxia (defined as those exhibiting a fold-change between hypoxic and normoxic conditions of <0.5 or >2) (Table S1). Notably, the distribution of miRNAs showed that more than half of the known hypoxamiRs (31 of 60, 51.67%) were presented in the DE miRNAs of cells after hypoxia, which indicated that hypoxia significantly modulated the hypoxamiRs expression in H9c2 cells (*p* = 9.99 × 10^−11^, χ^2^-test; Figure 2B). Additionally, we randomly selected seven miRNAs for validation of expression; the results demonstrated high correlation between the small RNA-seq and qRT-PCR (Figure 2C). Most of the DE miRNAs in cells have functions related to the cellular response to hypoxia. For example, miR-210-3p is known as the “master” hypoxamiR; it is induced by hypoxia in a HIF-dependent manner and has been shown to exhibit cardioprotective effects, such as preservation of cardiac function, upregulation of neovascularization, and inhibition of apoptosis [18]. Moreover, miR-351-5p directly target *Ang-2* and *VEGF* and has been shown to participate in hypoxia-induced microvascular dysfunction [19]. miR-199a-5p is acutely downregulated in cardiac myocytes after hypoxia and directly targets *HIF-1α* and *Sirtuin 1* to regulate the hypoxia-triggered pathway, it can be exploited for preconditioning cells against hypoxic damage [20].

### 2.3. Hypoxia Dramatically Alters the miRNAome of H9c2 Cells-Derived Exosomes

**Figure 3:**
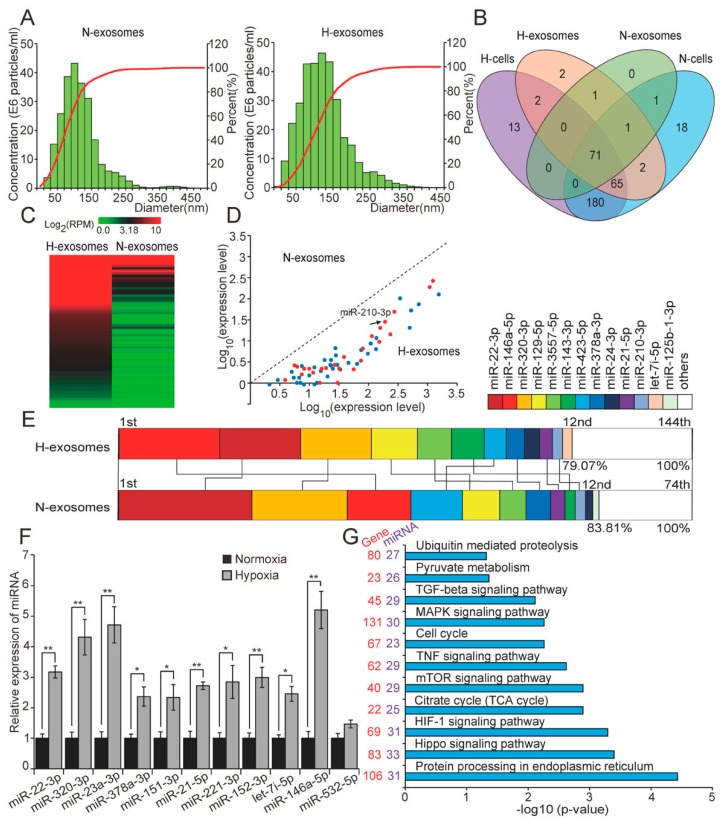
H9c2 cells-derived exosomal miRNAome was dramatically altered by hypoxia. (**A**) Particle size distribution in purified exosomes was measured by NanoSight Tracking Analyzer; (**B**) Venn diagram representing the numbers of miRNAs between H9c2 cells and exosomes before and after hypoxia; this revealed significant hypoxia-induced changes in the miRNAome of H9C2-derived exosomes; heatmap (**C**) and scatter diagram (**D**) showing the DE miRNAs (fold change <0.5 or >2 between hypoxia and normoxia) in exosomes; all of these DE miRNAs were upregulated in hypoxia compared with in normoxia; red dots represented known hypoxamiRs (arrow indicated miR-210-3p); (**E**) the 12 unique miRNAs with the highest expression levels in hypoxic and normoxic exosome miRNA libraries; this indicated that hypoxia significantly altered the ranking of the miRNAs within the list; (**F**) validation of small RNA-seq using qRT-PCR; and (**G**) gene ontology categories and pathways enriched for target genes of DE miRNAs in hypoxic exosomes. *p*-Values, which indicates the significance of the enrichment, were calculated by *Benjamini*-corrected modified Fisher’s exact test. “H” and “N” represented “Hypoxia” and “Normoxia”, respectively. All data are expressed as mean ± SD. * *p* < 0.05, ** *p* < 0.01.

Recent reports have shown that exosomes are produced by many cardiac-derived cell types, including cardiac fibroblasts [12], cardiac fibroblast-derived induced pluripotent stem cells [13], and cardiac progenitor cells [14]; these exosomes can play important roles in intercellular communication. We isolated exosomes from the culture media of H9c2 cells cultured under normoxic and hypoxic conditions by ultracentrifugation. The sizes distribution of the particles were investigated using NanoSight, a nanoparticle tracking analyser, which revealed particles ranging from 50 to 200 nm in diameter (Figure 3A), in keeping with typical exosome size [21,22]. Although the size range of exosomes were similar to normoxia group, the area under the curve of hypoxia group was increased, which indicated that hypoxia increased the number of exosomes secreted. Recent reports have suggested that some in vitro environmental stressors, such as heat [23], hypoxia [23,24], and glucose starvation [25], can enhance the secretion and modify the composition of exosomes in various cell types, including 3T3-L1 adipocytes [25], and cardiac progenitor cells [26]. Furthermore, we found that for both normoxia and hypoxia, total RNA isolated from H9c2 cells-derived exosomes contained a considerable number of small RNAs of approximately 25 nucleotides in length (Figure S1). These results showed that H9c2 cells produced miRNA-enriched exosomes under both normoxia and hypoxia.

Previously, Valadi et al. [11] suggested that miRNA-loaded exosomes can deliver their contents to other cell types and, thus, act as signaling mediators and exert functional regulation. To examine the miRNAome difference of H9c2 cells-derived exosomes under both normoxia and hypoxia, we conducted small RNA-seq analysis using total RNA isolated from the exosomes. This analysis identified 74 and 144 known miRNAs in normoxic and hypoxic H9c2 cells-derived exosomes, respectively. Notably, comparing the slight difference of miRNA species in cells, hypoxia manifestly increased miRNA species in exosomes (Figure 2A and Figure 3B). Interestingly, all of the DE miRNAs in exosomes (62 in total; fold-change <0.5 or >2; Table S2) were upregulated in hypoxia compared with in normoxia, and included a number of known hypoxamiRs (Figure 3C,D). These results showed that hypoxia altered both the types and expression levels of miRNAs in H9c2 cells-derived exosomes. Moreover, the 12 unique miRNAs with the highest expression levels accounted for 79.07% and 83.81% by total read counts of all miRNAs in hypoxic and normoxic exosomes, respectively. Meanwhile, 11 of the 12 most highly-expressed unique miRNAs were shared between normoxic and hypoxic exosomes, but their ranks in terms of expression levels obviously differed between the two conditions (Figure 3E). These results further demonstrated that hypoxia markedly changed the miRNAome of H9c2 cells-derived exosomes. Additionally, 11 miRNAs were randomly selected for qRT-PCR validation (Figure 3F).

To explore the potential functions of the DE miRNAs in hypoxic exosomes, we conducted target prediction and functional enrichment analysis using DIANA online software [27]. This revealed that the target genes of DE miRNAs in hypoxic exosomes were mainly involved in the HIF-1 signalling pathway, the cell cycle, or pathways involved in cell apoptosis, including the hippo, mTOR, TNF, MAPK, and TGF-β signalling pathways (Figure 3G), all of which are highly relevant to the hypoxic adaptability of cells. Notably, half of the DE miRNAs that we identified (31/62, 50%) were enriched in the HIF-1 signalling pathway; these hypoxamiRs directly target important mRNA transcripts that coordinate metabolic reprogramming, apoptosis, survival, and many other cellular processes that contribute to hypoxia adaptation. Additionally, some of DE miRNAs were enriched in functional processes related to cell viability under hypoxia, such as the TNF, MAPK, and mTOR signalling pathways. TNF family proteins, especially TNF-α, can activate multiple cell death pathways that contribute to progressive cardiomyocyte apoptosis [28,29]. The MAPK signalling pathway, which includes ERK1/2, JNK, and P38, plays an important role in cell proliferation, differentiation, and apoptosis [30]. Activation of JNK or p38 MAP kinases induces apoptosis in various cell types [31], including cardiomyocytes [32]. ERK1/2 exerts bidirectional effects in apoptosis, activation of ERK1/2, can activate or inhibit downstream effectors to determine cell fate [33]; acute activation of ERK1/2, exerts an anti-apoptotic effect and, thus, mitigates myocardial ischemia reperfusion injury (I/R injury), whereas chronic activation of ERK1/2 causes cardiomyocyte hypertrophy and is an essential mechanistic molecular pathway in adverse cardiac remodeling [34]. mTOR also has pleiotropic functions in the regulation of cell apoptosis; it can act as an inhibitor or an inducer of apoptosis depending on the cellular context [35]. Moreover, some of the DE miRNAs identified were enriched in the citrate cycle (the TCA cycle), pyruvate metabolism, ubiquitin mediated proteolysis, which suggests that hypoxia may affect the metabolism of glucose and some proteins.

### 2.4. Overexpression of Hypoxia-Induced Exosomal miRNAs Mitigates Hypoxia-Induced Apoptosis

**Figure 4:**
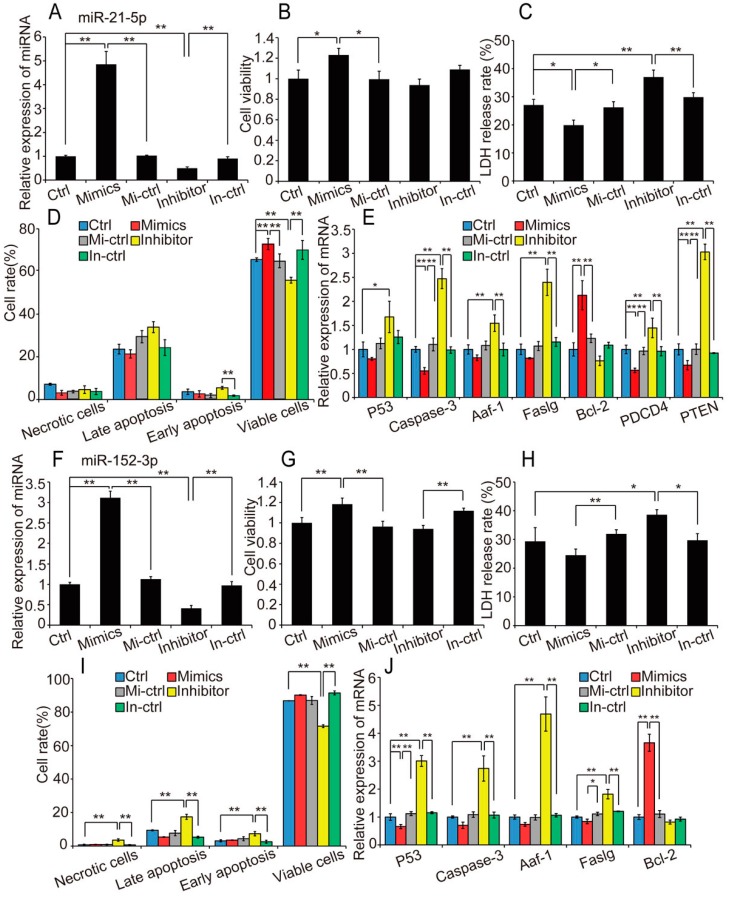
Overexpression of exosomal miRNA mitigates hypoxia-induced apoptosis. (**A**) Relative miRNA expression levels in control and miR-21-5p mimic- and inhibitor-transfected H9c2 cells; cells were exposed to hypoxia for 48 h after transfection; cell viability (**B**), membrane integrity (**C**) and apoptosis rate (**D**) were evaluated by CCK8, flow cytometry analysis and LDH release assay, respectively; expression levels of apoptosis-related genes and known target genes of miR-21-5p (*PTEN* and *PDCD4*) were measured by qRT-PCR (**E**); similarly, H9c2 cells were exposed to hypoxia 48 h after transfection with miR-152-3p and transfection efficiency (**F**); cell viability (**G**), membrane integrity (**H**), apoptosis rate (**I**) and apoptosis-related gene expression (**J**) were measured. “Ctrl”, “Mi-ctrl” and “In-ctrl” represented “control”, “mimics control”, and “inhibitor control”, respectively. Three independent experiments performed in triplicate and all data are expressed as mean ± SD. * *p* < 0.05, ** *p* < 0.01.

To further investigate the biological function of hypoxia-induced exosomal miRNAs in H9c2 cell apoptosis, we selected miR-21-5p, miR-378-3p, miR-152-3p, and let-7i-5p, which were significantly upregulated in hypoxic exosomes and participated in apoptosis, for functional verification. Among these miRNAs, miR-21-5p, and miR-378-3p have been reported to have anti-apoptotic effects in cardiomyocytes under hypoxic stress [36,37]. Gain- and loss-of-function analyses were performed using transfection of miRNA mimic (or mimic control), or inhibitor (or inhibitor control). qRT-PCR confirmed effective overexpression and downregulation of miRNAs in H9c2 cells transfected with the mimic and inhibitor, respectively (Figure 4A). Cells were exposed to hypoxia for 48 h after transfection, then cell viability, membrane integrity, and apoptosis rate were evaluated. As a result, under hypoxia condition, overexpression of miR-21-5p significantly suppressed the reduction of cell viability (*p* < 0.05; Figure 4B), reduced the cell membrane damage (*p* < 0.05; Figure 4C), and obviously promoted cell survival (Figure 4D). miR-21-5p overexpression significantly reduced the expression of the direct targets of miR-21 [38], *PTEN* and *PDCD-4*, and the apoptosis marker gene caspase-3, conversely, enhanced expression of the anti-apoptotic gene *Bcl-2* (Figure 4E). Similarly, upregulation of miR-378-3p robustly mitigated hypoxia-induced cell damage (Figure S2A–E). Consistent with previous research [36,38], our results suggest that upregulation of miR-21-5p and miR-378-3p mitigated hypoxia-induced apoptosis and enhanced cell viability, thereby supporting adaptation of the cell to the hypoxic conditions. 

Based on these findings, we focused on miR-152-3p and let-7i-5p for further study. We first transfected H9c2 cells with miRNA mimic (or mimic control), or inhibitor (or inhibitor control). Transfection efficiency is shown in Figure 4F. The experimental treatment and detection methods were similar to those described above. We found that miR-152-3p attenuated the reduction in cell viability (*p* < 0.01) and the increase in cell membrane damage (*p* < 0.05) induced by hypoxia in H9c2 cells (Figure 4G,H). Although the suppression of cell apoptosis induced by miR-152-3p upregulation was not significant (*p* = 0.056), miR-152-3p inhibition significantly reduced cell viability (*p* < 0.01) and increased rates of apoptosis and necrosis, which supports an anti-apoptotic effect of miR-152-3p (Figure 4I). Additionally, pro-apoptotic genes including *Caspase-3* and *Faslg* were inhibited by upregulation of miR-152-3p (Figure 4J). Similarly, let-7i-5p overexpression strongly reduced hypoxia-induced cell damage, including a reduction in cell membrane damage (*p* < 0.01) and apoptosis rate (*p* < 0.01), and downregulation of apoptosis-related genes (Figure S2F–J). These results demonstrate that miR-152-3p and let-7i-5p resemble miR-21-5p and miR-378-3p, which are highly expressed in hypoxic H9c2 cells-derived exosomes, and have anti-apoptotic and pro-viability effects in H9c2 cells under hypoxic stress.

### 2.5. Atg12 and Faslg Are Targets of miR-152-3p and Let-7i-5p, Respectively

**Figure 5:**
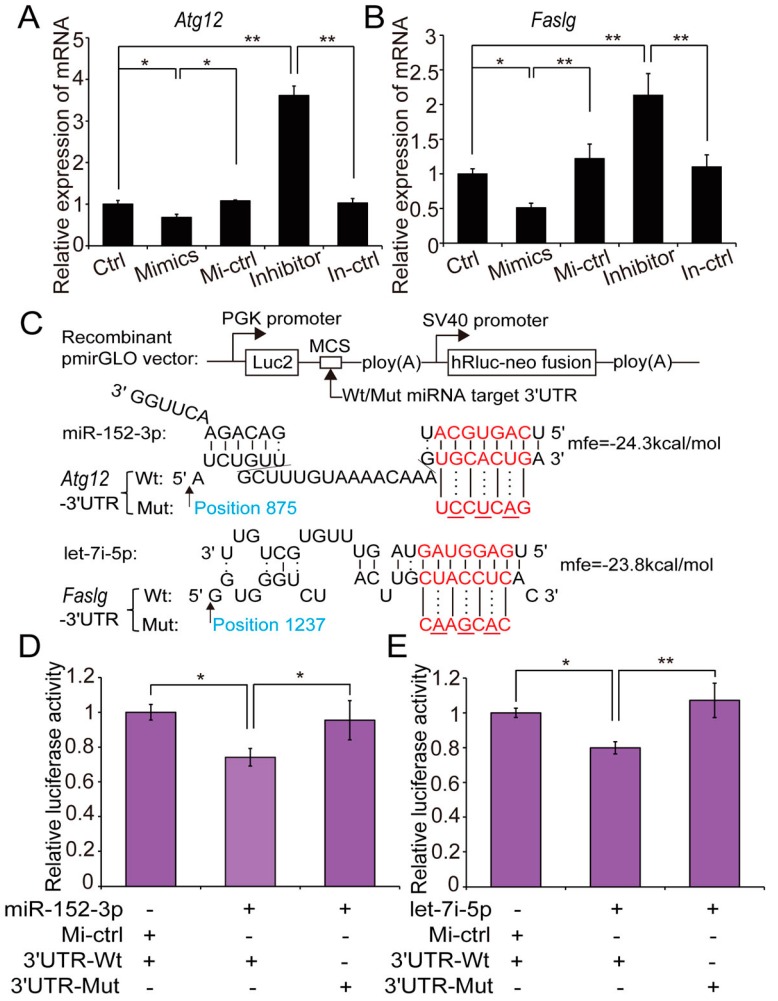
H9c2 cells-derived exosomal miRNAs exerted pro-viability effects by inhibiting apoptotic pathways. (**A**) Relative expression levels of *Atg12* mRNA after gain- and loss-of-function miR-152-3p transfection experiments, determined by qRT-PCR; the qRT-PCR data are normalized to *GAPDH*; (**C**) potential binding sites for miR-152-3p in the 3′-UTR of *Atg12* mRNA predicted by TargetScan, and the 3′-UTR *Atg12* mutation used in our study. A luciferase reporter assay was performed by co-transfecting a luciferase reporter containing the 3′-UTR of *Atg12* (wild-type [Wt] or mutant [Mut]) with a miR-152-3p mimic or control into HeLa cells; luciferase activity was determined 48 h after transfection (**D**); relative expression levels of *Faslg* mRNA after gain- and loss-of-function let-7i-5p transfection experiments (**B**); potential binding sites for let-7i let-7i-5p (**C**) and luciferase activity (**E**). “Ctrl”, “Mi-ctrl”, and “In-ctrl” represented “control”, “mimics control”, and “inhibitor control”, respectively. Three independent experiments performed in triplicate and all data are expressed as means ± SD. * *p* < 0.05, ** *p* < 0.01.

To explore the mechanism through which miR-152-3p and let-7i-5p mitigate hypoxia-induced H9c2 cells apoptosis, we predicted potential targets of miR-152-3p and let-7i-5p based on TargetScan and RNA hybrid analyses [39,40]. *Atg12* and *Faslg* were predicted as putative targets of miR-152-3p and let-7i-5p, respectively. *Atg12* encodes autophagy protein 12, a positive mediator of mitochondrial apoptosis that directly regulates the apoptotic pathway by binding and inactivating anti-apoptotic Bcl-2 family members, including Bcl-2 [41]. *Faslg* encodes Fas ligand, a member of the TNF family and the main activator of the extrinsic apoptotic pathway that binds the TNF receptor to induce apoptosis through the death-inducing signalling complex during heart failure [42], ischemia, and myocardial infarction [43]. *Atg12* and *Faslg* mRNA levels were reduced when miR-152-3p and let-7i-5p were overexpressed in hypoxic H9c2 cells (Figure 5A,B).

Bioinformatic prediction suggested that the 3′-UTR of *Atg12* mRNA has one unique putative binding site for the miR-152-3p seed sequence (Figure 5C). We constructed a dual luciferase report plasmid (pmirGLO-*Atg12* 3′-UTR) encoding the rat *Atg12* 3′-UTR that contains miR-152-3p binding sites. This reporter plasmid (with wild-type [Wt] or mutant [Mut] *Atg12* 3′-UTR) was co-transfected with miR-152-3p mimic into HeLa cells, and luciferase activity assay was performed 48 h after transfection. Upregulation of miR-152-3p significantly repressed luciferase activity in HeLa cells transfected with the wild-type *Atg12* 3′-UTR reporter (0.74-fold change, *p* = 0.017; Figure 5D), whereas this repression was completely abolished when the miR-152-3p binding site in *Atg12* was mutated. This indicates that miR-152-3p inhibited *Atg12* expression by binding the 3′-UTR. Similarly, cotransfection of pmirGLO-*Faslg* 3′-UTR (with Wt or Mut *Faslg* 3′-UTR) and let-7i-5p mimic into HeLa cells demonstrated that let-7i-5p was capable of targeting the 3′-UTR of Faslg mRNA (0.79-fold change in luciferase activity, *p* = 0.016; Figure 5E). These results indicate that *Atg12* and *Faslg* are the respective targets of miR-152-3p and let-7i-5p. miR-152-3p directly targeted *Atg12* and activated the anti-apoptotic gene *Bcl-2*, thus attenuating hypoxia-induced H9c2 cells apoptosis [41]; simultaneously, let-7i-5p played an anti-apoptotic role by inhibiting *Faslg* and, thus, attenuating the cell death-inducing signalling cascade [42,43].

Exosomes, which contain a spectrum of bioactive molecules including proteins, RNA, and lipids, have emerged as key mediators of intercellular communication [44]. Most cardiac cell types, including cardiomyocytes, cardiac fibroblasts, and cardiac progenitor cells, have been demonstrated to release exosomes that play an important role in improving cardiac function [22,45]. Recent reports have indicated that exosomes isolated from the blood have a cardioprotective effect, mitigating I/R injury [46], and that cross-species transfer of this cardioprotective effect is possible, with plasma from preconditioned pigs ameliorating I/R injury in rats [47]. In this study, we found that exosomes derived from H9c2 cells under hypoxic stress contained large amounts of cardioprotective miRNAs, which mitigated hypoxia-induced H9c2 cells apoptosis. We tentatively speculate a possible mechanism whereby, under hypoxic stress, hypoxia-sensitized H9c2 cells secrete and deliver hypoxamiR-enriched exosomes to surrounding cells to mitigate hypoxia-induced apoptosis and improve adaptation of the recipient cells to hypoxia. More specifically, we demonstrated for the first time that miR-152-3p and let-7i-5p, which were enriched in exosomes derived from H9c2 cells under hypoxic conditions, played an anti-apoptotic role via the mitochondrial (intrinsic) and the death receptor (extrinsic) pathways, respectively.

## 3. Materials and Methods

### 3.1. Cell Culture and Hypoxia Treatment

The rat cardiomyoblast cell line (H9c2) was obtained from the cell bank of the Chinese Academy of Sciences and cultured in DMEM medium (Hyclone, Logan, UT, USA) supplemented with 10% exosome-depleted FBS prepared by ultracentrifugation at 160,000× *g* at 4 °C for 16 h (Beckman, Brea, CA, USA). Normoxic cells were cultured in a humidified atmosphere of 95% air and 5% CO_2_ at 37 °C; The cells were subjected to hypoxic conditions for 48 h when they reached approximately 50% confluency. Hypoxic cells were cultured in the hypoxic chamber using AnaeroPack (Mitsubishi Gas Company, Tokyo, Japan) to mimic a hypoxic environment in vitro, with other conditions consistent with those of the normoxic cells. The AnaeroPack started to absorb oxygen within 1 min, oxygen tension inside the chamber dropped to 1 mmHg within 1 h (O_2_ < 1%, CO_2_ ≈ 5%) [48].

### 3.2. Exosome Isolaton

Exosomes were isolated from the conditioned medium of H9c2 cells by several centrifugation and filtration steps, as described previously [12,49], with some modifications. Briefly, 10 mL of conditioned medium were centrifuged at 300× *g* for 10 min, then at 2000× *g* for 10 min to remove cells and cellular debris; subsequently, the supernatant was further centrifuged at 10,000× *g* for 30 min at 4 °C and filtered through a 0.22-μm filter to remove subcellular structures. Finally, exosomes pellet were collected by ultracentrifugation at 160,000× *g* for 70 min. For further purification, the pellet were washed with PBS and centrifuged at 160,000× *g* for 70 min. The pellet was resuspended in 250 μL PBS, then immediately stored at −80 °C until use.

### 3.3. Nanoparticle Tracking Analysis

Nanoparticle tracking analysis (NTA) permits determination of both the size distribution and relative concentration of nanoparticles, by recording laser light scattered by particles in solution undergoing Brownian motion and, thus, tracking particle movement. Briefly, 300 μL pellet resuspensions were loaded into the sample chamber of an LM10 unit (Nanosight Technology, London, UK) and measurements were performed with a 405-nm 65-mW laser and an EMCCD Andor Luca camera. The analysis settings were optimized and kept identical each sample, and each video was analysed to give the mean, mode, median, and estimated concentration for each particle size. Data were analysed with NTA 2.1 software [50,51].

### 3.4. Small RNA Library Construction and Sequencing

Total RNA from H9c2 cells and corresponding exosomes were extracted using Trizol Reagent (Invitrogen, Carlsbad, CA, USA), according to the manufacturer’s instructions (*n* = 4). RNA quality was examined using an Agilent 2100 Bioanalyzer (Agilent Technologies, Redwood City, CA, USA). Small RNA-seq was conducted using an Illumina HiSeq 2500 platform (RiboBio, Guangzhou, China). Briefly, small RNA fractions ranging from 18 to 30 nucleotides were enriched from total RNA by 15% Tris-borate-EDTA polyacrylamide gel electrophoresis. To construct a small RNA sequencing library, 3′ and 5′ adaptors were ligated with the unique small RNA fractions. Subsequently, the adaptor-ligated RNA fragments were reverse-transcribed and amplified by PCR, and then were sequenced. The raw sequencing data have been submitted to the NCBI GEO database (GSE90123).

### 3.5. Prediction and Functional Annotation of Exosomal DE miRNA Targets

To explore the potential functions of the exosomal DE miRNAs, we conducted target prediction and functional enrichment analysis using DIANA-mirPath (http://www.microrna.gr/miRPathv3). DIANA-mirPath is a miRNA pathway analysis web-server, providing accurate statistics, while being able to accommodate advanced pipelines. mirPath can utilize predicted miRNA targets (in CDS or 3’-UTR) provided by the DIANA-microT-CDS algorithm, or even experimentally-validated miRNA interactions derived from DIANA-TarBase. These interactions (predicted and/or validated) can be subsequently combined with sophisticated merging and meta-analysis algorithms.

### 3.6. miRNA Transfection

Based on our small RNA-seq results and bioinformatic prediction, we selected several miRNA to use to verify function, including miR-21-5p, miR-378-3p, miR-152-3p, and miR-let-7i-5p. The specific miRNA mimic and inhibitor were purchased from RIBOBIO (Guangzhou, China). H9c2 cells were transfected using Lipofectamine 2000 (Invitrogen) according to the manufacturer’s instructions. In brief, cells were transfected when they reached 50% confluency. Five groups of cells were prepared: control (no transfection); mimic-transfected; mimic-transfected control (transfected with miRNA containing no sequence similarities to any reported rat gene sequence); inhibitor-transfected (transfected with a single RNA sequence exactly complementary to the specific miRNA mimic); and inhibitor-transfected control. Transfection complexes were prepared by a series of premixing steps according to the manufacturer’s protocol, and were then added to the medium to a final concentration of 50 nM (or 100 nM in the inhibitor-transfected group). Six hours after transfection, the medium was replaced. All groups were then exposed to hypoxia treatment for 48 h until the following experiment.

### 3.7. Detection of Viability and Cell Damage by CCK8 and LDH Release

To evaluate H9c2 cells survival and membrane integrity after exposure to hypoxia, cell viability and LDH release were measured by CCK8 and LDH Release Assay Kit (Beyotime Biotechnology, Shanghai, China), respectively. CCK8 could react with dehydrogenase in active cells to form formazan. At 1.5–2 h before analysis, 10 μL CCK8 was added to the medium of cells cultured in 96-well plates. Optical density was measured at 450 nm using a microplate reader (Thermo, Madrid, Spain). Cell viability is presented as a percentage of control absorbance (that of untransfected cells) after subtracting the background absorbance of cell-free culture medium. LDH activity in the culture medium was determined with a microplate reader (at 490 nm) using an enzymatic rate method. LDH release rate was calculated as a percentage of the maximum enzymatic activity of a control sample.

### 3.8. Flow Cytometry Analysis of Cell Apoptosis

Cell apoptosis rates were detected by flow cytometry using an Annexin V apoptosis detection kit (BD Pharmingen, San Diego, CA, USA). PI (propidium iodide) is commonly used for identifying dead cells and is generally excluded from viable cells. AV (Annexin V-FITC) is a common marker of apoptosis because of its high affinity for phosphatidylserine, which is translocated from the inner to the outer leaflet of the plasma membrane early in apoptosis. After 48 h of hypoxia treatment, cells were trypsin-digested and collected by centrifugation at 1000× *g* for 5 min. Each sample was double-stained for 10 min with AV and PI and then analyzed by flow cytometry. Cells stained with AV and PI or AV only were considered necrotic and apoptotic, respectively.

### 3.9. Fluorescence Staining of H9c2 Cells with Hoechst 33342/PI

Cells were washed twice with PBS then stained with Hoechst 33342 (10 mg/mL) for 10 min. The cells were then washed with PBS again and stained with PI for 10 min. Stained cells were imaged by fluorescence microscopy (Olympus, Tokyo, Japan).

### 3.10. Dual-Luciferase Activity Assay

The potentially targeted mRNA contained the specific miRNA binding sites (wild-type [Wt] or mutant [Mut]) were synthesized from TSINGKE (Chengdu, China). The sequences were cleaved using Sac I/Xho I and cloned into the pmirGLO plasmid (Promega Corporation, Madison, WI, USA) at the 3′-end of the firefly luciferase reporter (*luc2*) gene (Figure 5C). HeLa cells were cultured in 96-well plates, when the cell density reached about 80% confluence, recombinant pmirGLO vector with [Wt] or [Mut] co-transfected with miRNA mimics into cells by Lipofectamine3000 (Invitrogen). Cells were collected after 48 h, dual-luciferase activity was measured using the Dual-Luciferase Reporter Assay System kit (Promega), according to the manufacturer’s instructions.

### 3.11. qRT-PCR

Total RNA (including miRNA) was extracted with TRIzol Reagent (Invitrogen). mRNA and miRNA were reverse-transcribed respectively using PrimeScript RT reagent Kit with gDNA Eraser and Mir-X™ miRNA First Strand Synthesis Kit (Takara, Dalian, China), following the manufacturer’s recommendations. qPCR was performed using an SYBR Premix Ex Taq kit (Takara) and a CFX96 system (Bio-Rad, Hercules, CA, USA). All reactions were performed in triplicate. Relative expression levels of mRNAs and miRNAs were calculated using the 2^−ΔΔ*C*t^ method. *GAPDH* and *U6* were used as housekeeping genes for normalizing mRNA and miRNA, respectively. Sequences of the primers used for qPCR are shown in Table S3.

### 3.12. Statistical Analysis

All data are expressed as mean ± SD. Statistical significance was calculated by one way analysis of variance (ANOVA) with Tukey’s post-hoc test for multiple groups or Student’s *t*-test for comparisons of two groups, using SPSS 19.0 software (SPSS Inc., Chicago, IL, USA). *p* < 0.05 was considered statistically significant (* *p* < 0.05; ** *p* < 0.01).

## 4. Conclusions

In this study, we revealed the miRNAome of H9c2 cells and exosomes under both hypoxia and normoxia. We identified 331, 338, 144, and 74 unique mature miRNAs in hypoxic cells, normoxic cells, hypoxic exosomes, and normoxic exosomes, respectively. Moreover, we identified 92 and 62 DE miRNAs in cells and exosomes between hypoxia and normoxia, respectively. These exosomal DE miRNAs were mainly involved in the HIF-1 signaling pathway or pathways related to cell apoptosis, such as the TNF, MAPK, and mTOR pathways. Interestingly, we found that some exosomal DE miRNAs, including miR-21-5p, miR-378-3p, miR-152-3p, and let-7i-5p, had potential anti-apoptotic and pro-viability effects in H9c2 cells under hypoxic stress. Additionally, we identified *Atg12* and *Faslg* as the respective targets of miR-152-3p and let-7i-5p, which partly elucidates the anti-apoptotic mechanism of hypoxia-induced exosomal miRNA. In brief, our results reveal that exosomes derived from H9c2 cells in response to hypoxia loaded with large amounts of cardioprotective miRNAs and mitigate hypoxia-induced H9c2 cells apoptosis, which may present a potential novel treatment for AMI and other types of heart disease.

## Supplementary Materials

Supplementary materials can be found at www.mdpi.com/1422-0067/18/4/711/s1.

## Abbreviations

DE miRNA	Differentially-Expressed miRNA
AMI	Acute Myocardial Infarction
HIF	Hypoxia-Inducible Factor
HRE	Hypoxia Response Elements
CPCs	Cardiac Progenitor Cells
CCK8	Cell Counting Kit-8
LDH	Lactate Dehydrogenase
NTA	Nanoparticle Tracking Analysis
FBS	Foetal Bovine Serum
PBS	Phosphate-Buffered Saline
AV	Annexin V-FITC
PI	Propidium Iodide
