# Supplementary material for: Overexpression of Exosomal Cardioprotective miRNAs Mitigates Hypoxia-Induced H9c2 Cells Apoptosis
Source: Int J Mol Sci. 2017 Mar 28;18(4):711. doi: 10.3390/ijms18040711 (PMC5412297; doi:10.3390/ijms18040711)
Supplement: Supplementary file 1 [file ijms-18-00711-s001.docx]

*Supplementary material*

Overexpression of Exosomal Cardioprotective miRNAs Mitigates Hypoxia-Induced H9c2 Cells Apoptosis

Jinwei Zhang^†^, Jideng Ma^†^, Keren Long^†^, Wanling Qiu, Yujie Wang, Zihui Hu, Can Liu, Yi Luo, Anan Jiang, Long Jin, Qianzi Tang, Xun Wang, Xuewei Li*, Mingzhou Li*

Institute of Animal Genetics and Breeding, College of Animal Science and Technology, Sichuan Agricultural University, Chengdu, Sichuan 611130, China; [Jinweizhang50@163.com](mailto:Jinweizhang50@163.com) (J.Z.); [jideng_ma@sina.com](mailto:jideng_ma@sina.com) (J.M.); [longkeren@163.com](mailto:longkeren@163.com) (K.L.); [qiuwanling2016@163.com](mailto:qiuwanling2016@163.com) (W.Q.); [wangyujie715@163.com](mailto:wangyujie715@163.com) (Y.W.) ; [Huzihui2016@163.com](mailto:Huzihui2016@163.com) (Z.H.); [18227550880@163.com](mailto:18227550880@163.com) (C.L.); luoyi670@163.com(Y.L.); lingdang317@163.com(A.J.); longjin8806@163.com(L.J.); wupie@163.com(Q.T.); [xun_wang007@163.com](mailto:xun_wang007@163.com) (X.W.); xuewei.li@sicau.edu.cn (X.L.); mingzhou.li@163.com (M.L.)

* Author to whom correspondence should be addressed; E-Mail: mingzhou.li@163.com (M.L.); xuewei.li@sicau.edu.cn (X.L.); Tel.: +86-28-8629-0962 (M.L.); +86-28-8629-0998 (X.L.); Fax: +86-28-8629-0962 (M.L.); +86-28-8629-0998 (X.L.).

†These authors contributed equally to this work.

**Supplementary Figure S1.** Agilent 2100 TapeStation analysis of total RNA from H9c2 cells and exosomes.

**Supplementary Figure S2.** Anti-apoptotic effects of almost exosomal miRNA was revealed by gain- and loss- function experiments.

**Supplementary Table S1.** DE miRNAs in H9c2 cells

**Supplementary Table S2.** DE miRNAs in H9c2 cells-derived exosomes.

**Supplementary Table S3.** List of gene-specific primers used for qRT-PCR


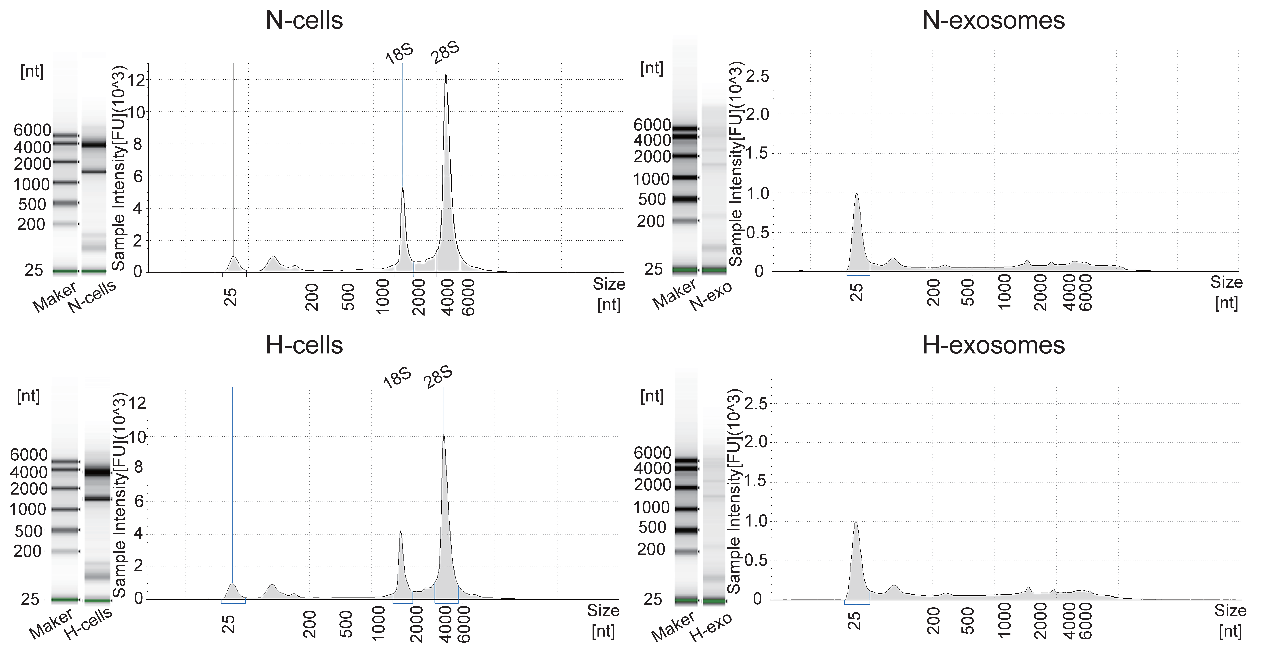


**Supplementary Figure S1.** Agilent 2100 TapeStation analysis of total RNA from H9c2 cells and exosomes. Gels and electropherograms are shown. The left gel lanes represent an RNA ladder standard and the right lanes represent the total RNA from the sample. The y-axes of the electropherograms represent arbitrary fluorescence unit intensity (FU) and the x-axes indicate RNA fragment size (in nucleotides).


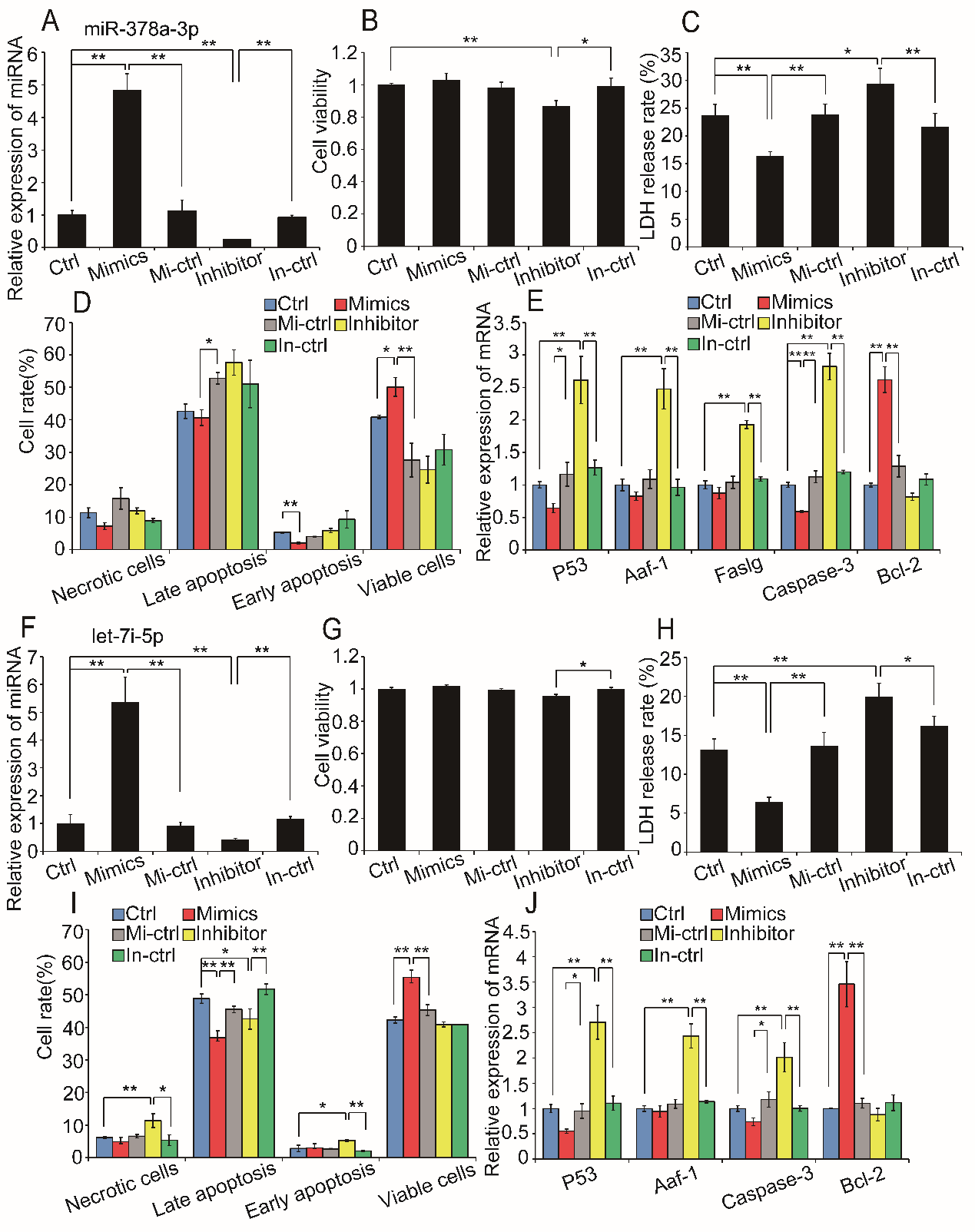


**Supplementary Figure S2. Anti-apoptotic effects of some exosomal miRNA was revealed by gain- and loss- function experiments. (A)** Transfection efficiency of miR-378 was measured by qRT-PCR. H9c2-transfected cells were exposed to hypoxia for 48 h, then cell viability **(B)**, membrane integrity **(C)** and apoptosis rate **(D)** were evaluated by CCK8, flow cytometry analysis and LDH release assay, respectively. Expression levels of apoptosis-related genes and the known miR-378 target gene *Caspase-3* were measured by qRT-PCR **(E)**. Similarly, transfection efficiency **(F)**, cell viability **(G)**, membrane integrity **(H)**, apoptosis rate **(I)**, and apoptosis-related gene expression **(J)** were measured in let-7i-transfected cells after 48 h of exposure to hypoxic conditions. “Ctrl”, “Mi-ctrl” and “In-ctrl” represented “control”, “mimics control” and “inhibitor control”, respectively. Three independent experiments performed in triplicate and all data are expressed as mean ± SD. **P* <0.05, ***P* < 0.01.

**Supplementary Table S1** DE miRNAs in H9c2 cells

| **No.** | **miRNA_ID** | **Hypoxic Cells (RPM)** | **Normoxic Cells**  **(RPM)** | **Fold-change Hypoxia/Normoxia** |
| --- | --- | --- | --- | --- |
| *1* | *rno-miR-30b-3p* | *9.0326* | *18.1646* | *0.497263909* |
| *2* | *rno-let-7e-3p* | *2.9356* | *5.9588* | *0.492649527* |
| *3* | *rno-miR-24-1-5p* | *3.0861* | *6.3432* | *0.486520999* |
| *4* | *rno-miR-191a-5p* | *2116.4843* | *4397.7501* | *0.48126525* |
| *5* | *rno-miR-146a-3p* | *3.0109* | *6.3432* | *0.474665784* |
| *6* | *rno-miR-29b-3p* | *86.4117* | *184.1446* | *0.469260027* |
| *7* | *rno-miR-21-5p* | *130725.1605* | *280136.5246* | *0.466648041* |
| *8* | *rno-miR-107-3p* | *54.4966* | *119.3672* | *0.456545852* |
| *9* | *rno-let-7b-5p* | *2046.0302* | *4493.0901* | *0.455372618* |
| *10* | *rno-let-7c-5p* | *2441.8078* | *5392.7653* | *0.452793264* |
| *11* | *rno-miR-130a-3p* | *20.5491* | *46.3245* | *0.443590325* |
| *12* | *rno-let-7i-3p* | *7.9788* | *18.2607* | *0.436938343* |
| *13* | *rno-miR-188-5p* | *2.6345* | *6.0549* | *0.435102149* |
| *14* | *rno-miR-224-3p* | *1.2796* | *3.0755* | *0.416062429* |
| *15* | *rno-miR-181a-1-3p* | *52.0879* | *125.5182* | *0.414982847* |
| *16* | *rno-miR-92a-1-5p* | *2.2581* | *5.4782* | *0.412197437* |
| *17* | *rno-miR-199a-5p* | *5117.7852* | *12467.3398* | *0.410495365* |
| *18* | *rno-miR-26b-3p* | *1.0538* | *2.5949* | *0.406104281* |
| *19* | *rno-miR-122-5p* | *5.7206* | *14.8969* | *0.384012781* |
| *20* | *rno-miR-27a-5p* | *102.4445* | *267.5671* | *0.382874053* |
| *21* | *rno-let-7b-3p* | *11.2155* | *29.7938* | *0.37643738* |
| *22* | *rno-miR-30d-3p* | *6.5486* | *17.9724* | *0.364369811* |
| *23* | *rno-miR-30e-5p* | *510.4914* | *1421.5464* | *0.35910991* |
| *24* | *rno-miR-23b-5p* | *1.5807* | *4.421* | *0.357543542* |
| *25* | *rno-miR-148a-5p* | *18.1404* | *50.8416* | *0.356802304* |
| *26* | *rno-miR-125b-1-3p* | *156.8658* | *459.3043* | *0.341529134* |
| *27* | *rno-miR-181b-1-3p* | *3.3119* | *12.4942* | *0.265074995* |
| *28* | *rno-miR-21-3p* | *30.786* | *119.3672* | *0.257910046* |
| *29* | *rno-miR-146b-3p* | *5.4948* | *22.3934* | *0.24537587* |
| *30* | *rno-miR-146b-5p* | *714.5524* | *3009.6495* | *0.23742047* |
| *31* | *rno-miR-7a-5p* | *770.9308* | *3754.0128* | *0.205361793* |
| 32 | rno-miR-542-5p | 3.3872 | 1.0572 | 3.203934922 |
| 33 | rno-miR-217-5p | 29.4312 | 10.3798 | 2.835430355 |
| 34 | rno-miR-411-5p | 2.3334 | 0.9611 | 2.427843096 |
| 35 | rno-miR-450b-3p | 30.2591 | 13.1669 | 2.298118768 |
| 36 | rno-miR-331-3p | 11.1402 | 22.2973 | 0.49962103 |
| 37 | rno-miR-361-3p | 65.4862 | 131.8614 | 0.496629036 |
| 38 | rno-miR-140-3p | 1160.3103 | 2347.2671 | 0.494323931 |
| 39 | rno-miR-25-5p | 1.7312 | 3.556 | 0.486839145 |
| 40 | rno-miR-877 | 2.5592 | 5.286 | 0.484146803 |
| 41 | rno-miR-499-5p | 12.7209 | 26.6222 | 0.477830532 |
| 42 | rno-miR-99a-5p | 14.7532 | 31.1393 | 0.473780721 |
| 43 | rno-miR-452-5p | 172.5976 | 365.406 | 0.472344734 |
| 44 | rno-miR-532-5p | 481.1355 | 1019.523 | 0.471922164 |
| 45 | rno-miR-455-5p | 74.3683 | 158.003 | 0.470676506 |
| 46 | rno-miR-342-5p | 0.9033 | 1.9222 | 0.469930288 |
| 47 | rno-miR-301a-5p | 6.5486 | 14.0319 | 0.466693748 |
| 48 | rno-miR-676 | 113.1331 | 242.4827 | 0.466561532 |
| 49 | rno-miR-18a-5p | 12.194 | 26.2377 | 0.464751102 |
| 50 | rno-miR-10a-5p | 5715.3658 | 12330.673 | 0.463508018 |
| 51 | rno-miR-193b-3p | 3.2367 | 7.0159 | 0.461337818 |
| 52 | rno-miR-206-3p | 1.5054 | 3.2677 | 0.460691006 |
| 53 | rno-miR-3596a | 1.5054 | 3.2677 | 0.460691006 |
| 54 | rno-miR-3473 | 12.4198 | 27.0066 | 0.459880177 |
| 55 | rno-miR-125a-3p | 7.6024 | 16.6268 | 0.457237713 |
| 56 | rno-miR-1306-3p | 1.4302 | 3.1716 | 0.450939589 |
| 57 | rno-miR-221-5p | 99.5089 | 223.7415 | 0.444749409 |
| 58 | rno-miR-324-5p | 5.8712 | 13.3591 | 0.439490684 |
| 59 | rno-miR-219a-1-3p | 3.0109 | 6.9198 | 0.435113732 |
| 60 | rno-miR-130a-5p | 1.5054 | 3.4599 | 0.43509928 |
| 61 | rno-miR-6319 | 0.828 | 1.9222 | 0.430756425 |
| 62 | rno-miR-10b-5p | 444.9299 | 1072.3829 | 0.414898354 |
| 63 | rno-miR-598-3p | 16.1834 | 39.4046 | 0.410698243 |
| 64 | rno-miR-3120 | 97.4013 | 244.0204 | 0.399152284 |
| 65 | rno-miR-3549 | 1.2796 | 3.2677 | 0.391590415 |
| 66 | rno-miR-99b-3p | 71.7337 | 183.568 | 0.390774536 |
| 67 | rno-miR-362-5p | 38.0121 | 99.1844 | 0.383246761 |
| 68 | rno-miR-32-5p | 8.6562 | 22.8739 | 0.378431313 |
| 69 | rno-miR-874-3p | 4.3657 | 11.6292 | 0.375408455 |
| 70 | rno-miR-142-3p | 0.828 | 2.2105 | 0.374575888 |
| 71 | rno-miR-191a-3p | 2.4087 | 6.4393 | 0.374062398 |
| 72 | rno-miR-132-5p | 1.3549 | 3.7482 | 0.361480177 |
| 73 | rno-miR-219a-5p | 2.3334 | 6.6315 | 0.351866094 |
| 74 | rno-miR-9b-3p | 11.4413 | 33.1576 | 0.345058147 |
| 75 | rno-miR-30c-1-3p | 3.0861 | 9.1303 | 0.338006418 |
| 76 | rno-miR-145-3p | 24.0116 | 72.9466 | 0.329166815 |
| 77 | rno-miR-203a-3p | 3.0861 | 9.4187 | 0.327656683 |
| 78 | rno-miR-3559-3p | 8.0541 | 24.7 | 0.326076923 |
| 79 | rno-miR-9a-5p | 8.1293 | 25.7572 | 0.315612722 |
| 80 | rno-miR-425-5p | 47.4211 | 154.2547 | 0.307420779 |
| 81 | rno-miR-128-1-5p | 1.3549 | 4.5171 | 0.299949082 |
| 82 | rno-miR-582-5p | 6.8497 | 24.1233 | 0.283945397 |
| 83 | rno-miR-582-3p | 53.0664 | 191.1606 | 0.277601137 |
| 84 | rno-miR-129-5p | 987.788 | 3737.0015 | 0.264326359 |
| 85 | rno-miR-193a-3p | 0.828 | 3.1716 | 0.261066969 |
| 86 | rno-miR-27b-5p | 9.71 | 38.3474 | 0.25321143 |
| 87 | rno-miR-760-3p | 7.3013 | 29.5054 | 0.247456398 |
| 88 | rno-miR-196a-3p | 3.9141 | 17.2035 | 0.227517656 |
| 89 | rno-miR-152-5p | 11.2907 | 52.4754 | 0.215161771 |
| 90 | rno-miR-615 | 5.1185 | 24.4117 | 0.20967405 |
| 91 | rno-miR-33-5p | 2.8603 | 15.3774 | 0.186006737 |
| 92 | rno-miR-671 | 1.5054 | 10.2836 | 0.146388424 |

Note: Red italic represent “known hypoxamiRs”

**Supplementary Table S2** DE miRNAs in H9c2 cells-derived exosomes

| **No.** | **miRNA_ID** | **Hypoxic Exosomes (RPM)** | **Normoxic**  **Exosomes (RPM)** | **Fold-change Hypoxia/Normoxia** |
| --- | --- | --- | --- | --- |
| *1* | *rno-miR-22-3p* | *1236.0954* | *266.7169* | *4.634484729* |
| *2* | *rno-miR-320-3p* | *1087.6345* | *189.7777* | *5.731097489* |
| *3* | *rno-miR-378a-3p* | *274.8228* | *49.2984* | *5.574679908* |
| *4* | *rno-miR-24-3p* | *235.0767* | *14.2418* | *16.50610878* |
| *5* | *rno-miR-21-5p* | *192.1736* | *28.5678* | *6.72693032* |
| *6* | *rno-miR-210-3p* | *158.7415* | *20.3935* | *7.783926251* |
| *7* | *rno-let-7i-5p* | *152.9941* | *9.5226* | *16.06642094* |
| *8* | *rno-miR-125b-1-3p* | *114.6242* | *12.9777* | *8.832397112* |
| *9* | *rno-miR-152-3p* | *80.3017* | *6.5731* | *12.21671662* |
| *10* | *rno-miR-23a-3p* | *74.7971* | *3.3708* | *22.18971757* |
| *11* | *rno-miR-214-3p* | *56.4216* | *2.1068* | *26.78071008* |
| *12* | *rno-miR-148a-3p* | *38.6128* | *4.1293* | *9.350931151* |
| *13* | *rno-miR-27a-3p* | *32.1369* | *2.191* | *14.66768599* |
| *14* | *rno-miR-186-5p* | *30.0322* | *1.0112* | *29.69956487* |
| *15* | *rno-miR-30d-5p* | *26.7942* | *2.6967* | *9.935921682* |
| *16* | *rno-miR-181a-5p* | *19.185* | *2.2753* | *8.43185514* |
| *17* | *rno-miR-181b-5p* | *16.1089* | *2.191* | *7.352304884* |
| *18* | *rno-miR-148b-3p* | *14.9756* | *1.0112* | *14.80973101* |
| *19* | *rno-miR-27b-3p* | *11.5757* | *1.7697* | *6.541052156* |
| *20* | *rno-let-7c-5p* | *9.9568* | *2.191* | *4.544408946* |
| *21* | *rno-let-7a-5p* | *8.1759* | *2.191* | *3.731583752* |
| *22* | *rno-miR-199a-5p* | *7.4473* | *1.6011* | *4.651364687* |
| *23* | *rno-miR-192-5p* | *6.2331* | *2.4439* | *2.550472605* |
| *24* | *rno-let-7g-5p* | *5.5855* | *2.6124* | *2.138072271* |
| *25* | *rno-miR-101a-3p* | *3.8856* | *1.1798* | *3.293439566* |
| 26 | rno-miR-146a-5p | 1551.15 | 127.4174 | 12.17376905 |
| 27 | rno-miR-129-5p | 709.7633 | 74.4111 | 9.538406232 |
| 28 | rno-miR-3557-5p | 527.3036 | 53.1748 | 9.916419056 |
| 29 | rno-miR-143-3p | 498.6475 | 20.5621 | 24.25080609 |
| 30 | rno-miR-423-5p | 343.4678 | 102.8946 | 3.33805467 |
| 31 | rno-miR-221-3p | 136.2376 | 11.7136 | 11.63071985 |
| 32 | rno-miR-151-3p | 134.2948 | 6.4046 | 20.9684914 |
| 33 | rno-miR-140-3p | 109.1196 | 8.6799 | 12.57152732 |
| 34 | rno-miR-532-5p | 97.8677 | 4.972 | 19.68376911 |
| 35 | rno-miR-199a-3p | 75.4447 | 6.0675 | 12.43423156 |
| 36 | rno-miR-30a-5p | 64.1928 | 2.8652 | 22.40429987 |
| 37 | rno-miR-10a-5p | 45.8173 | 3.0337 | 15.10277878 |
| 38 | rno-miR-146b-5p | 33.3511 | 2.1068 | 15.83021644 |
| 39 | rno-miR-145-5p | 29.7084 | 2.6967 | 11.01657581 |
| 40 | rno-let-7b-5p | 28.1703 | 4.5506 | 6.190458401 |
| 41 | rno-miR-100-5p | 27.6037 | 6.7417 | 4.094471721 |
| 42 | rno-miR-3556a | 24.9324 | 2.191 | 11.37946143 |
| 43 | rno-miR-423-3p | 23.2324 | 3.5394 | 6.563937391 |
| 44 | rno-miR-185-5p | 23.1515 | 2.6124 | 8.862157403 |
| 45 | rno-miR-25-3p | 22.6658 | 2.6967 | 8.405013535 |
| 46 | rno-miR-147 | 20.3183 | 1.4326 | 14.18281446 |
| 47 | rno-miR-486 | 18.0517 | 3.5394 | 5.100214726 |
| 48 | rno-miR-99b-5p | 15.6232 | 1.9382 | 8.060674853 |
| 49 | rno-miR-1839-5p | 13.4376 | 1.0112 | 13.28876582 |
| 50 | rno-miR-760-3p | 12.1424 | 2.3596 | 5.145956942 |
| 51 | rno-miR-151-5p | 12.1424 | 0.927 | 13.09859763 |
| 52 | rno-miR-582-3p | 9.9568 | 1.5169 | 6.563913244 |
| 53 | rno-miR-222-3p | 8.0949 | 0.927 | 8.73236246 |
| 54 | rno-miR-125b-5p | 7.4473 | 2.5281 | 2.945809106 |
| 55 | rno-miR-24-2-5p | 7.0426 | 1.0112 | 6.964596519 |
| 56 | rno-miR-328a-3p | 6.7188 | 1.3483 | 4.983163984 |
| 57 | rno-miR-92a-3p | 6.1521 | 1.0112 | 6.083959652 |
| 58 | rno-miR-127-3p | 5.4236 | 1.3483 | 4.022546911 |
| 59 | rno-miR-339-5p | 5.0189 | 1.854 | 2.707065804 |
| 60 | rno-miR-99a-5p | 2.9142 | 1.1798 | 2.470079675 |
| 61 | rno-miR-193a-5p | 2.1047 | 0.927 | 2.270442287 |
| 62 | rno-miR-3586-5p | 1.8618 | 0.8427 | 2.209327163 |

Note: Red italic represent “known hypoxamiRs”

**Supplementary Table S3** List of gene-specific primers used for qRT-PCR

| **Gene name** | **Sequence(5' to 3')** |
| --- | --- |
| *Caspase-3* | F- CATGGCCCTGAAATACGAAGTC  R- GCAGGCCTGAATGATGAAGAGTTT |
| *PTEN* | F- CCAGTCAGAGGCGCTATGTA  R- TACATGAGCTTGTCCTCCCG |
| *PDCD4* | F- AGGTTGCTAGATAGGCGGTC  R-GTCTTCTCAAACGCCGTCTC |
| *Faslg* | F- GCCCGTGAATTACCCATGTC  R- TAGTGGTGATGGAGGTGGTG |
| *P53* | F- CTCCTCTCCCCAGCAAAAGA  R- GTAGACTGGCCCTTCTTGGT |
| *Atg12* | F- TGTCCAAGCACTCATCGACT  R- TCATCCCCATGCCTGTGATT |
| *Aaf-1* | F- CGCTTAACATTGAGGAGGCC  R- TCCCTAGACCACTCTCCACA |
| *Bax* | F- TGGCCTCCTTTCCTACTTCG  R- AAAATGCCTTTCCCCGTTCC |
| *Bcl-2* | F- GACGCGAAGTGCTATTGGT  R- TCAGGCTGGAAGGAGAAGAT |
| *miR-210-3p*  *miR-351-5p*  *miR-7a-5p*  *miR-199a-5p*  *miR-450b-3p*  *miR-146a-5p*  *miR-107-3p*  *miR-22-3p*  *miR-320-3p*  *miR-23a-3p* *miR-378a-3p* *miR-21-5p*  *miR-221-3p*  *miR-151-3p*  *let-7i-5p*  *miR-152-3p*  *miR-146a-5p* *miR-532-5p* | F- CTGTGCGTGTGACAGCGGCTGA  F-TCCCTGAGGAGCCCTTTGAGCCTGA  F- TGGAAGACTAGTGATTTTGTTGT  F- CCCAGTGTTCAGACTACCTGTTC  F- ATTGGGGACGCTTCGCATTCA  F-TGAGAACTGAATTCCAUGGGTT  F-AGCAGCATTGTACAGGGCTATCA  F-AAGCTGCCAGTTGAAGAACTGT  F-AAAAGCTGGGTTGAGAGGGCGA  F- ATCACATTGCCAGGGATTTCC  F- ACTGGACTTGGAGTCAGAAGG  F- TAGCTTATCAGACTGATGTTGA  F- AGCTACATTGTCTGCTGGGTTTC  F- CTAGACTGAGGCTCCTTGAGG  F- TGAGGTAGTAGTTTGTGCTGTT  F- TCAGTGCATGACAGAACTTGG  F- TGAGAACTGAATTCCATGGGTT  F- CATGCCTTGAGTGTAGGACTGT |
| *GAPDH* | F- AACGACCCCTTCATTGACCTC  R- CCTTGACTGTGCCGTTGAACT |
| *U6* | F-CTCGCTTCGGCAGCACA  R-AACGCTTCACGAATTTGCGT |
|  |  |

Note: GAPDH and U6 were used as housekeeping genes for normalising mRNA and miRNA, respectively.
